# Changes in Kidney Fat upon Dietary-Induced Weight Loss

**DOI:** 10.3390/nu14071437

**Published:** 2022-03-30

**Authors:** Manuela Spurny, Yixin Jiang, Solomon A. Sowah, Tobias Nonnenmacher, Ruth Schübel, Romy Kirsten, Theron Johnson, Oyunbileg von Stackelberg, Cornelia M. Ulrich, Rudolf Kaaks, Hans-Ulrich Kauczor, Tilman Kühn, Johanna Nattenmüller

**Affiliations:** 1 Department of Diagnostic and Interventional Radiology, Heidelberg University Hospital, 69120 Heidelberg, Germany; manuela.spurny@web.de (M.S.); peggy_jiang@hotmail.com (Y.J.); tobias.nonnenmacher@med.uni-heidelberg.de (T.N.); ruth.schuebel@gmx.de (R.S.); oyunbileg.stackelberg@med.uni-heidelberg.de (O.v.S.); hans-ulrich.kauczor@med.uni-heidelberg.de (H.-U.K.); 2 German Cancer Research Center (DKFZ), Division of Cancer Epidemiology, 69120 Heidelberg, Germany; sowahsolo@yahoo.com (S.A.S.); t.johnson@dkfz-heidelberg.de (T.J.); r.kaaks@dkfz-heidelberg.de (R.K.); t.kuhn@qub.ac.uk (T.K.); 3 Biobank of the National Center for Tumor Diseases (NCT) Heidelberg, 69120 Heidelberg, Germany; romy.kirsten@nct-heidelberg.de; 4 Department of Population Health Sciences, Huntsman Cancer Institute, University of Utah, Salt Lake City, UT 84112-5550, USA; neli@hci.utah.edu; 5 Institute for Global Food Security (IGFS), Queen’s University Belfast, Belfast BT9 5DL, UK; 6 Heidelberg Institute of Global Health (HIGH), Heidelberg University Hospital, 69120 Heidelberg, Germany; 7 Department of Diagnostic and Interventional Radiology, Medical Center-University of Freiburg, Faculty of Medicine, University of Freiburg, 79106 Freiburg, Germany

**Keywords:** magnetic resonance imaging, kidney fat content, renal sinus fat, diet induced weight loss, obesity, overweight, body composition

## Abstract

As the metabolic role of kidney fat remains unclear, we investigated the effects of dietary weight loss on kidney fat content (KFC) and its connection to kidney function and metabolism. Overweight or obese participants (*n* = 137) of a dietary intervention trial were classified into quartiles of weight loss in a post hoc manner. Kidney sinus (KSF) and cortex fat (KCF) were measured by magnetic resonance imaging at baseline, week 12 and week 50. Weight loss effects on KFC were evaluated by linear mixed models. Repeated measures correlations between KFC, other body fat measures and metabolic biomarkers were obtained. KSF, but not KCF, decreased significantly across weight loss quartiles at week 12 (quartile 4: −21.3%; *p* = 0.02) and 50 (−22.0%, *p* = 0.001), which remained significant after adjusting for VAT. There were smaller improvements regarding creatinine (−2.5%, *p* = 0.02) at week 12, but not week 50. KSF, but not KCF, correlated with visceral (r_rm_ = 0.38) and subcutaneous fat volumes (r_rm_ = 0.31) and liver fat content (r_rm_ = 0.32), as well as diastolic blood pressure and biomarkers of lipid, glucose and liver metabolism. Dietary weight loss is associated with decreases in KSF, but not KCF, which suggests that KSF may be the metabolically relevant ectopic fat depot of the kidney. KSF may be targeted for obesity-related disease prevention.

## 1. Introduction

Overweight (Body Mass Index (BMI) ≥ 25–30 kg/m^2^) and obesity (BMI ≥ 30 kg/m^2^) are major health problems of the modern society, that are not restricted to industrial, wealthy countries, but also affect low- and middle-income countries [1,2]. Over the last decades, the prevalence of overweight and obesity have increased tremendously worldwide [3]. Health consequences of obesity are an increased risk for the metabolic syndrome [4], cardiovascular diseases [5] and also different types of cancer [6]. Worldwide, diseases caused by obesity lead to high costs, not just in the health care system [7,8], but also in the economy [9,10]. Therefore, a major interest of health politics is to support obese and overweight people losing weight to avoid medical and economic consequences [11].

Obesity causes ectopic accumulation of adipose tissue in physiologically not designated locations of the body after the physiological storage of fat such as the subcutaneous adipose tissue (SAT) is exceeded [12]. Organs and tissues such as the liver, the heart, the pancreas, the muscle, the bone marrow and also the kidneys account for ectopic fat depots [12,13]. Within the kidney there are two possible locations for fat depots: the kidney sinus (i.e., renal sinus) and the kidney cortex (i.e., renal-parenchymal) [13,14,15]. Moreover, the kidneys are surrounded by the retroperitoneal adipose tissue within the Gerota’s fascia, which is part of the visceral adipose tissue (VAT) compartment. The kidney sinus fat is located within the hilum of each kidney, with contact to the external renal capsule, and thus a peri-renal fat depot [14,16]. High amounts of kidney sinus fat have been associated with hypertension, atherosclerosis, visceral obesity and chronic kidney disease [14,16,17]. One possible mechanism for hypertension and its secondary diseases as a consequence of increased accumulation of kidney sinus fat might be the activation of the renin angiotensin aldosterone system (RAAS) caused by a compression of the hilar structures such as veins, arteries, lymphatics and ureters due to fat accumulation [16,18]. Regarding the kidney cortex fat, which is composed of fat accumulations within the kidney parenchyma, rare data exist with no clear pathological link in human studies, but hints in animal studies at causing hypertension and kidney damage [14,15,19,20].

While kidney fat, and especially kidney sinus fat, seems to be connected with the pathogenesis of obesity-related diseases, only few studies exist on the question whether pathophysiological consequences of renal fat accumulation are reversible, and only one weight loss trial has been carried out so far [14]. Therefore, it is of great importance to gain a better understanding of kidney fat and its connections with metabolic function and lipid accumulation in other metabolically active body fat depots. In particular, it is interesting if kidney fat is an independent metabolic risk factor from VAT, as it remains unclear whether ectopic fat accumulation generally exerts metabolic effects beyond those of the metabolically active VAT [21]. Moreover, it is important to assess whether specific risks associated with excess accumulation of kidney fat could be reduced via weight loss in early phases of overweight and obesity to avoid potentially irreversible long-term cardio-metabolic impairments.

The aim of this trial was to examine whether kidney fat content decreases after weight loss in a dietary intervention study among metabolically healthy adult obese or overweight individuals. Additionally, we analysed if the kidney fat content is associated with other body fat depots such as VAT, SAT, liver fat content (LFC) and pancreatic fat content (PFC) as well as metabolic biomarkers. To this end, we used data originating from a randomized controlled trial (RCT), initially designed to assess the metabolic effects of intermittent vs. continuous calorie restriction, in a post hoc manner, using overall weight loss achieved during the trial to create a model on the effects of weight loss on kidney fat and related biomarkers. Whole body Magnetic Resonance Imaging (MRI)-examinations were performed at baseline, after 12 weeks of intervention and after 50 weeks to measure the kidney fat content as well as the aforementioned fat depots.

## 2. Material and Methods

### 2.1. Study Population

For this study, data from the HELENA-Trial (trial registration number: NCT02449148 ClinicalTrials.gov), a randomized dietary intervention study, which had been undertaken at the German Cancer Research Centre (DKFZ), Heidelberg, and at the Heidelberg University Hospital from May 2015 to May 2017, was used to investigate the effects of continuous vs. intermittent calorie restriction in a post hoc manner.

The study included 150 obese or overweight individuals (50% female), who were non-smokers and aged between 35 and 65 years. Exclusion criteria were severe chronic diseases, which comprised kidney or liver dysfunction, major cardiovascular diseases and cancer. Additionally, diabetes mellitus, HbA1c levels ≥ 6.5% and/or fasting glucose levels ≥ 126 mg/dL measured at screening were defined as exclusion criteria. At baseline, the participants were assigned either to the group with intermittent calorie restriction, to that with continuous calorie restriction or to the control group. The study consisted of a 12-week intervention phase, a 12-week maintenance phase and a 26-week follow-up phase. At the beginning, all participants had given their written informed consent, and the study was approved by the responsible ethics committee at Heidelberg University Hospital, Germany (Vote S-299/2014).

Each participant received three MRI examinations, the first at baseline, the second after 12 weeks of intervention and the last one after 50 weeks. The exclusion criteria of the MRI examinations included claustrophobia, cardiac pacemakers or defibrillators, non-removable, medical and/or electronic foreign bodies, which were not approved for 1.5 Tesla-MR, joint protheses or metallic foreign bodies. Out of the initial 150 participants, six did not participate in the intervention phase and in seven participants, MRI analyses for kidney fat content were not available. Thus, for the present study, 137 participants were included.

At baseline, after 12 weeks, after 24 weeks, and after 50 weeks, participants filled out questionnaires, underwent medical examinations and provided blood samples. Biweekly phone calls by dietary assistants during the intervention phase were performed to record side effects and the compliance of the participants.

Further details on the rationale and design of the HELENA-Trial can be found in previous publications [22,23].

### 2.2. Laboratory Methods

The blood-based biomarkers glucose, HDL (high-density lipoprotein), cholesterol, triglycerides and HbA1c were measured in the central laboratory of Heidelberg University Hospital, Heidelberg, Germany. Serum biomarkers were analysed by electrochemiluminiscence on a Quickplex SQ 120 (Meso Scale Discoveries, Rockville, MD, USA) in the Division of Cancer Epidemiology at the German Cancer Research Centre (DKFZ), Heidelberg, Germany. Estimated GFR (glomerular filtration rate) was calculated by the CKD-EPI formula [24], which is the most accurate equation in respect of obese people [25]. A detailed description of the laboratory analyses can be found in a previous publication [22].

### 2.3. Imaging

Kidney fat content (KFC), i.e., fat content of kidney sinus and kidney cortex, liver fat content (LFC), pancreas fat content (PFC), visceral adipose tissue (VAT) and subcutaneous adipose tissue (SAT) were measured with a 1.5 Tesla MR scanner (MAGNETOM Aera, Siemens Healthcare, Erlangen, Germany) by generating a thoracoabdominal 2-point DIXON sequence and an abdominal multi-echo GRE sequence. Hardware, software and MR protocol remained constant for all MRI examinations. Details of imaging and MR protocol are given in the study design paper by Schübel et al. [23].

Fat content of cortex and sinus of both kidneys was measured by a multi-echo GRE (gradient echo) technique (Siemens LiverLab, Siemens Healthcare, Erlangen, Germany) [23,26,27]. Regions of interest (ROI; area = 0.5 cm^2^) were positioned manually in the cortex and sinus of the right and left kidney on the proton density fat fraction (PDFF) map using a post-processing software (OsiriX, Pixmeo SARL, Bernex, Switzerland) [27]. The placement of the ROIs avoided the borders of the organ, vessels, possible lesions, artefacts and lymph nodes. The right kidney is often positioned at a lower abdominal level than the left kidney. Therefore, in some cases, the ROIs of the right kidney needed to be placed at a lower slice than those of the left kidney. The MRI measurements of each participant were performed at baseline, after 12 weeks, and after 50 weeks with a constant positioning of each ROI for each time point.

Intra- and inter-reader coefficients were assessed in 40 examinations and were 0.93 and 0.89, respectively (Readers MS and YJ).

Liver fat content was assessed with three regions of interest of 4 cm^2^ each, which were placed separately in the dorsal, the anterior-medial, and the anterior-lateral right liver lobe directly above the porta hepatis on a PDFF map with the same post-processing software as for the KFC (OsiriX, Pixmeo SARL, Bernex, Switzerland). Details of LFC measurements are published previously [28,29].

Pancreas fat content was manually measured by placing 3 ROIs (each 0.785 cm^2^) in the pancreas head, body, and tail within the PDFF map derived from the multi-echo GRE sequence by using the same post-processing software tool (OsiriX, Pixmeo SARL, Bernex, Switzerland). Details of PFC measurements have been published previously [21].

The measurements of VAT and SAT were performed with a 2-point Dixon sequence from neck to the upper part of the legs and a semi-automatic quantification with an in-house developed software based on the Medical Imaging Interaction Toolkit (MITK, DKFZ, Heidelberg, Germany) was applied [30].

### 2.4. Statistical Analyses

The primary hypothesis of the HELENA trial was that intermittent calorie restriction is superior to continuous calorie restriction with respect to metabolism, adipose tissue gene expression, and body composition, which was disproved by previously published results showing no significant differences between both calorie restriction types across primary, secondary, and exploratory endpoints [22,31]. Therefore, the study cohort was re-classified in a post hoc manner into weight loss quartiles irrespective of the weight loss method. Weight loss quartiles were constructed based on the amount of weight lost after the 12-week intervention phase as follows: Quartile 1 (Q1) (≤2%, *n* = 35), quartile 2 (Q2) (>2 and ≤4.5%, *n* = 34), quartile 3 (Q3) (>4.5% and ≤7.5%, *n* = 35), and quartile 4 (Q4) (>7.5%, *n* = 33). These equally large quartiles were utilized to obtain a larger statistical power in comparison to a priori determined cut-off points. Importantly, there were no statistically significant differences between the study groups at baseline with respect to kidney fat content and other body composition and metabolic parameters used for the present post analyses on overall weight loss.

To determine the associations between kidney fat parameters and body composition, anthropometric parameters as well as metabolic parameters over all time points, repeated measures correlations (rmcorr) with the R package (The R foundation for Statistical Computing, Vienna, Austria) were used [32]. Rmcorr is a statistical technique for analysing the common within-subject association for paired variables assessed at two or more time points for a group of individuals [32]. The technique produces an rmcorr coefficient (r_rm_) between −1 and 1, similar to that produced by the Pearson correlation coefficient which indicates the strength of the linear association between two variables, while simultaneously accounting for non-independence of the repeated measurements. The kidney fat parameters and all other parameters were log-transformed prior to the repeated measures correlation analyses. The *p* values from the repeated measures correlation analyses were corrected for multiple testing using the Bonferroni correction method [33] and *p* values less than 0.05 after Bonferroni correction (for three tests, i.e., kidney cortex fat, kidney sinus fat and total kidney fat) were considered statistically significant. Thus, only *p* values less than 0.0167 (i.e., 0.05/3) prior to Bonferroni correction remained significant after correction.

Additionally, Spearman’s coefficients were calculated to assess the association of KFC with anthropometric parameters, blood pressure, blood biochemistry markers (markers of glucose and lipid metabolism, kidney and liver function test, inflammation and adipokines), and body composition parameters (VAT, SAT, LFC and PFC) for each time point separately (see Supplementary Materials Table S1).

The effect of overall weight loss on changes in kidney cortex fat, kidney sinus fat, total kidney fat and kidney function parameters, i.e., serum creatinine and estimated glomerular filtration rate (eGFR) was evaluated using linear mixed models. Trends for linear associations between weight loss and changes in each of the aforementioned variables, i.e., kidney fat and kidney function parameters were modelled with weight loss (weight change) as a continuous parameter. The linear mixed model included age, sex, time, weight change, and a time-by-weight change interaction term, with participants’ identifier or subject ID set as the random effect. Associations with a time-by-weight change interaction term less than 0.05, i.e., *p*-interaction < 0.05 were considered significant. The kidney fat and kidney function parameters were log-transformed prior to the linear mixed models analyses. Additionally, the linear mixed model was adjusted for VAT at both time points.

SAS 9.4 (Cary, NC, USA) was used for the statistical analyses.

## 3. Results

### 3.1. Characteristics of the Study Population at Baseline

The characteristics of the study population at baseline are shown in Table 1. Parts of the baseline data have already been published by this group [21,34]. Overall, kidney cortex fat contents, kidney sinus fat and total kidney fat contents were similarly distributed across the four quartiles at baseline, while the absolute values were higher in the kidney total and kidney sinus fat content than in the kidney cortex fat content (see Table 1). Height, weight, BMI, SAT, and VAT were similarly distributed across all quartiles at baseline. Participants in Q4 were slightly younger (47.3 ± 8.5 years) than the participants in Q1, Q2 and Q3 (50.9 ± 6.4 years, 50.8 ± 8.4 years, 51.2 ± 7.9 years). Notably, however, neither this difference nor differences in any other parameters at baseline were statistically significant according to ANOVA tests.

### 3.2. Correlations of Kidney Fat Content with Blood Biomarkers, Blood Pressure, Anthropometrics and Body Fat Depots

**Figure 1:**
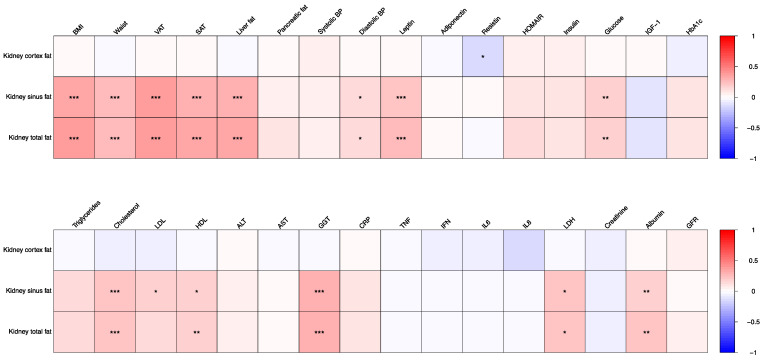
Repeated measures correlations of kidney cortex fat, kidney sinus fat and kidney total fat over all time points (time points T0, T1, and T3 combined). The asterisks *, ** and *** represent *p*-values (after Bonferroni correction) of 0.05, 0.01 and 0.001, respectively. Abbreviations: LDL, low density lipoprotein, SAT, subcutaneous adipose tissue; BMI, body mass index; VAT, visceral adipose tissue, ALT, alanine aminotransferase; AST, aspartat aminotransferase, GGT, gamma glutamyl transferase, CRP, C-reactive protein, TNF, tumour necrosis factor, IFN, interferon, IL, Interleukin, LDH, lactate dehydrogenase.

Correlations of total kidney fat content, renal cortex fat content and renal sinus fat content with anthropometrics (waist circumference and BMI), blood pressure, body fat parameters, and blood biomarkers over all three time points combined (baseline, after 12 and 50 weeks) are given in Table 2 and in Figure 1. Spearman‘s correlations for the aforementioned parameters at each time point (baseline, after 12 and 50 weeks) separately are given in Supplementary Materials Table S1.

Regarding BMI, a moderate positive correlation for kidney sinus fat and total kidney fat, with a r_rm_ of 0.35 and 0.36, respectively, and a *p* < 0.0001 for both was observed. Regarding waist circumference, a correlation with a r_rm_ of 0.26 and a *p* < 0.0001 both for kidney sinus fat and total kidney fat was found.

In correlation analyses on body fat parameters, a stronger significant correlation between kidney sinus fat as well as total kidney fat and VAT, with a r_rm_ of 0.38 and 0.39, respectively, and a *p* < 0.0001 for each was found. For SAT a significant correlation with kidney sinus fat and total kidney fat (r_rm_: 0.31 and 0.33; *p* < 0.0001 both) was observed. For liver fat content also a significant correlation with kidney sinus fat and total kidney fat (r_rm_: 0.32 both; *p* < 0.0001 both) was seen. There was no significant correlation between kidney cortex fat and VAT, SAT and liver fat content. Pancreas fat content showed no significant correlation with any kidney fat content parameter.

With respect to blood pressure, a weak, but statistically significant correlation between diastolic blood pressure and both kidney sinus fat and total kidney fat was seen (r_rm_: 0.15 and 0.16). No significant correlation was seen for kidney cortex fat and blood pressure.

Of the liver function tests, only GGT showed a correlation with kidney sinus fat and total kidney fat (r_rm_: 0.31 and 0.32, <*p* = 0.0001 for both).

Regarding kidney function parameters, albumin correlated weakly, but significantly with both kidney sinus fat and total kidney fat (r_rm_: 0.19 and 0.20). Creatinine and the GFR showed no significant correlation with any kidney fat parameter.

Statistically significant, but weaker, correlations were found with the further parameters such as leptin, resistin, HOMA-IR, glucose, triglycerides, cholesterol, HDL and LDH.

The associations observed at each individual time points using Spearman‘s correlations (see Supplementary Materials Table S1) were to a considerable extent similar to those observed using repeated measures correlations. Interestingly, some body fat parameters showed an even stronger correlation: between VAT and kidney sinus fat (rho: 0.45, 0.44, and 0.55 for baseline, week 12 and 50) and total kidney fat (rho: 0.46, 0.46, and 0.56 for baseline, week 12 and 50). Additionally, pancreas fat content, which did not show any significant correlation in the repeated measures correlations, exhibited significant correlations with both kidney sinus fat (rho: 0.27, 0.29, and 0.33 for baseline, week 12 and 50) and total kidney fat (rho: 0.27, 0.31, and 0.33 for baseline, week 12 and 50). These slightly stronger correlations obtained when using Spearman’s coefficients rather than rmcorr coefficients may be due to slightly skewed distributions of some of the used parameters, even after log-transformation.

### 3.3. Effects of Weight Loss on Kidney Fat Content, Creatinine, and Glomerular Filtration Rate (GFR)

**Figure 2:**
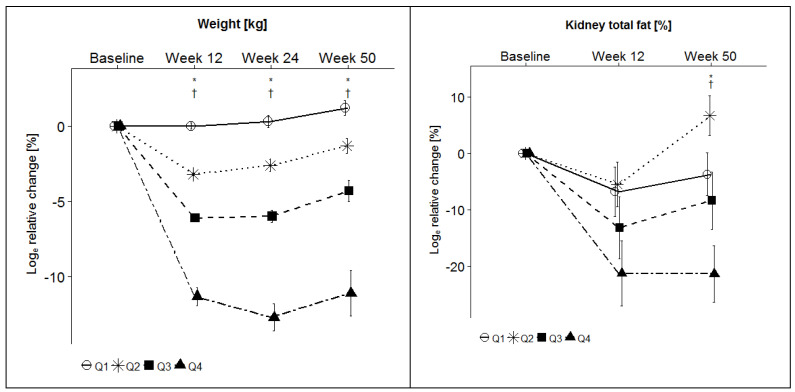

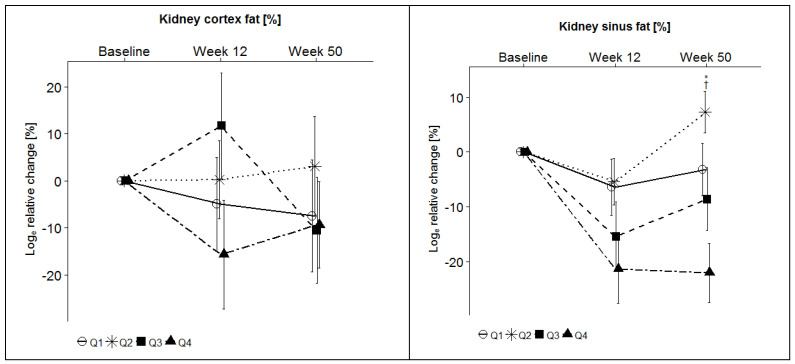
Relative changes of weight, total kidney fat content, kidney cortex fat content and kidney sinus fat content, *n* = 137. Data are shown as mean ± SEM for Log relative change. The baseline values serve as reference. Abbreviations: SEM, standard error of the mean; †, significant between the extreme quartiles 1 and 4; *, significant for all quartiles. The figure of weight change was published previously [21,34].

Relative changes of body weight, total kidney fat content, renal cortex fat content, renal sinus fat content, creatinine and GFR from baseline to week 12 and from baseline to week 50 are shown in Table 3 and in Figure 2. The data on body weight have already been published in two previous papers by this group [21,34].

The relative changes in body weight from baseline to week 12 was 0.0 ± 0.2% for Q1, −3.3 ± 0.1% for Q2, −6.1 ± 0.2% for Q3 and −11.5 ± 0.6% for Q4. At week 50, the relative changes of body weight were 1.3 ± 0.6% for Q1, −1.3 ± 0.5% for Q2, −4.4 ± 0.8% for Q3 and −11.2 ± 1.6% for Q4 [21,34].

Relative changes in total kidney fat content from baseline to week 12 were −6.8 ± 4.4% for Q1, −5.5 ± 4.0% for Q2, −13.2 ± 5.5% for Q3 and −21.3 ± 5.8% for Q4 (*p* = 0.01), from baseline to week 50 −3.7 ± 3.8% for Q1, 6.7 ± 3.5% for Q2, −8.4 ± 5.1% for Q3 and −21.4 ± 5.0% for Q4 (*p* <0.0001), with a significant decrease for each time point across all quartiles (*p* = 0.01 and *p* = 0.0001). Relative changes in renal cortex fat content were from baseline to week 12 −5.0 ± 10.0 for Q1, 0.2 ± 8.3 for Q2, 11.7 ± 11.2 for Q3 and −15.7 ± 11.6 for Q4 (*p* = 0.97), from baseline to week 50 −7.5 ± 11.9 for Q1, 3.1 ± 10.6 for Q2, −10.5 ± 11.3 for Q3 and −9.4 ± 9.2 for Q4 (*p* = 0.77), with no significant decrease for each time point across all quartiles. For renal sinus fat content, relative changes were from baseline to week 12 −6.4 ± 5.1 for Q1, −5.4 ± 4.2 for Q2, −15.4 ± 6.3 for Q3 and −21.3 ± 6.3 for Q4 (*p* = 0.02), and from baseline to week 50 −3.2 ± 4.8 for Q1, 7.3 ± 3.8 for Q2, −8.6 ± 5.7 for Q3 and −22.0 ± 5.4 for Q4 (*p* = 0.001), with a significant decrease for each time point across all quartiles (*p* = 0.02 and *p* = 0.001). After adjusting for changes in VAT, the aforementioned significant changes in total kidney fat and kidney sinus fat persisted; thus, the observed changes in the kidney fat parameters are independent of changes in VAT.

Weight loss was significantly associated with changes in plasma creatinine concentrations at week 12 (Q1: 2.8 ± 1.4, Q2: 2.0 ± 1.3, Q3: 3.4 ± 1.3, Q4: −2.5 ± 1.8, *p* = 0.02), although the differences induced by weight loss were very moderate, and not significant at week 50 (Q1: 3.0 ± 1.6, Q2: −1.1 ± 1.2, Q3: 4.8 ± 1.4, Q4: 2.2 ± 2.0, *p* = 0.77). Similarly, a significant, but very moderate effect of weight loss on GFR was observed at week 12 (−1.0 ± 0.5, Q2: −0.8 ± 0.5, Q3: −0.8 ± 0.8, Q4: 1.1 ± 0.7, *p* = 0.04), but not from baseline to week 50 (Q1: −1.0 ± 0.6, Q2: 0.8 ± 0.7, Q3: −2.1 ± 0.7, Q4: −0.8 ± 0.8, *p* = 0.65).

## 4. Discussion

In this present work, the impact of dietary-induced weight loss on kidney fat content among overweight or obese individuals, as well as correlations between kidney fat content and markers of metabolism was studied. The main finding of the present study is that kidney fat content (sinus and total fat) decreased significantly after 12 weeks of intervention and remained diminished after the follow-up phase at week 50 depending on weight loss. These changes were independent of changes in VAT. Simultaneously, creatinine and GFR as kidney function parameters improved after the intervention phase, which, however, did not sustain after the follow-up phase.

The decrease in kidney fat was observed for the parameters kidney sinus fat and total kidney fat, but not in kidney cortex fat content (i.e., intra-parenchymal fat accumulation). As the total kidney fat content is composed of kidney sinus fat and cortex fat, the decrease in kidney fat seems to be driven by the kidney sinus fat content. These findings were in line with results of a long-term (18 months) weight loss trial by Zelicha et al. including 278 participants with abdominal obesity and dyslipidemia [14]. In this study, a significant linear decrease in kidney sinus fat across quintiles of weight loss was observed, whereas decreases in kidney cortex fat (i.e., parenchymal fat) were weaker and did not show a strong linear trend [14]. Interestingly, Zelicha et al. showed that the association between overall weight loss and kidney sinus fat in their trial was not differential across the trial arms, i.e., a low-fat diet vs. a low-carbohydrate Mediterranean diet. Given the distinct nutrient composition of these diets, it seems conceivable that calorie restriction as such, rather than the underlying dietary pattern, affects kidney sinus fat in humans. In our study, calorie reduction was mostly due to a moderate reduction in calories from fat in both initial trial arms [22]. Otherwise, Zelicha et al. also showed simultaneous decreases in visceral fat, liver fat and pancreatic fat as well as improvements in a broader range of cardio-metabolic biomarkers and blood pressure with weight loss, similar as in the present study [14,21,29]. Although it is difficult to disentangle in a human weight loss trial which metabolic improvements are attributable to reductions in specific ectopic fat depots and VAT, these data are in line with a central role of a ‘fatty kidney’ as a driver of metabolic dysfunction in obesity [35,36]. They may also point to a strong preventive potential of weight loss interventions among patients with overweight with regard to chronic kidney disease [35].

Similar as VAT and LFC, kidney fat content showed a linear decrease with weight loss in the present study [21]. This is in contrast to previous findings by us and others on pancreatic fat content and bone marrow fat, indicating that PFC and bone marrow fat only decrease with a more pronounced weight loss [21,34]. After adjusting for changes in VAT, the aforementioned changes in total kidney fat and kidney sinus fat persisted; thus, the observed changes in the kidney fat parameters are independent of changes in VAT, which is in line with results of Zelicha et al. [14]. This is an important finding as pancreas fat content, for example, which is also an abdominal fat depot, is not independent from VAT in some studies and also in the present cohort [21].

After the 12 weeks of intervention, creatinine and GFR as kidney function parameters improved significantly, albeit modestly, which, however, did not sustain after the follow-up phase. These results are in line with a small, short term dietary intervention study by Giordani et al. in obese patients with diabetes mellitus, who observed an improvement of the GFR after seven days of very low-calorie diet [37]. However, Motie et al. observed no change of kidney function parameters after a three month weight loss intervention in 59 obese patients with heart failure, diabetes mellitus and/ or metabolic syndrome, but with normal renal function [38]. Additionally, a previous study by von Scholten et al. did not note any improvement in renal function after moderate weight loss, whereat the amounts of weight loss were similar to the present study (6% to 8%) [39]. Thus, results of weight loss trials regarding kidney functions are quite different from each other. One reason may be the grade of obesity and different co-morbidities, but also the chosen type of weight loss intervention (hard and fast diet versus slow weight reduction over weeks). An alternative weight loss approach used among morbidly obese people is bariatric surgery. There are several studies on kidney function after bariatric surgery [25,40,41,42,43] showing that extreme weight reduction by bariatric surgery can prevent chronic kidney disease [41]. While a decrease in GFR may be due to a decrease in hyperfiltration after severe weight loss [43], the development of GFR after surgery also seems to depend on the kidney function before bariatric-surgery-induced weight loss [43,44]. The participants of the HELENA-Trial had no severe kidney diseases and were only obese or overweight, but not of extreme obesity at baseline; thus, our results cannot be compared easily. Furthermore, the degree of weight loss after bariatric surgery is typically greater than the dietary-induced weight loss such as in the HELENA-Trial.

With regard to correlations of kidney fat (total and sinus fat) with other body fat depots, the strongest significant correlation (repeated measure over all three time points) was found with VAT (0.39), followed by SAT (0.33) and liver fat content (0.32). By using Spearman’s correlations, VAT (rho = 0.55), SAT (rho = 0.29) and LFC (rho = 0.35) correlated even stronger with kidney fat (total and sinus), and also PFC (rho = 0.33) showed significant correlations (see Supplementary Materials Table S1), which is in line with the study by Zelicha et al. [14]. Additionally, Notohamiprodjo et al. found a correlation of kidney sinus fat and VAT with increasing kidney volume and sinus fat in pre-diabetic and diabetic individuals in comparison to individuals with normal glucose levels [45]. However, kidney cortex fat showed no significant correlations with these parameters, which was also seen in a study by Sijens et al. with normal weight and obese subjects, in which kidney cortex fat did not correlate with SAT, PFC and LFC [15].

Regarding kidney function parameters, only albumin was weakly correlated with sinus (0.19) and total kidney fat (0.20), but not GFR or creatinine. Zelicha et al. also reported no correlation between GFR and kidney fat content [14].

With regard to blood pressure, which decreased significantly after weight loss (for both diastolic and diastolic blood pressure across the weight loss quartiles after 12 and 50 weeks after intervention, see previous publications by this group [22]), only a weak correlation of sinus (0.15) and total kidney fat (0.16) with diastolic blood pressure was observed. Zelicha et al. could not observe a correlation in a similar study setting [14]. However, Chughtai et al. found that the amount of kidney sinus fat is correlated with hypertension and, interestingly, with the number of antihypertensive medications in patients with cardiovascular risk factors [16]. As Chughtai et al. did not differentiate between elevated blood pressure because of diastolic or systolic blood pressure, the results cannot be compared thoroughly. Thus, in synopsis with the above-mentioned literature, this weak correlation should be considered with caution.

Regarding metabolic biomarkers, a stronger correlation of sinus and total kidney fat (up to 0.33) with GGT as liver enzyme was found, which was also seen by Zelicha et al. [14]. However, the other liver enzymes (ALT and AST) did not show any significant correlation. Other weak correlations were seen for sinus and total kidney fat with markers of glucose metabolism (glucose, but not HOMA-IR, insulin, HbA1c, IGF-1), lipid metabolism (cholesterol, HDL, LDL, but not triglycerides), leptin and LDH, but not for markers of inflammation, which partly corresponds to the results by Zelicha et al. [14], who described similar results after 18 months. As these weak correlations with metabolic markers show no consistent pattern, these results should be considered with caution. Again, kidney cortex fat showed no correlation with any metabolic parameter with the exception of resistin.

The correlations of kidney sinus fat with other body fat depots, anthropometrics, markers of metabolism and also clinical markers, such as blood pressure, support, that the kidney sinus fat is the metabolic relevant ectopic fat depot of the kidney, and therefore these results are in line with the literature, in which kidney sinus fat is associated with a number of obesity-related morbidities such as hypertension, chronic kidney disease or arthrosclerosis [14,16,17]. On the contrary, kidney cortex fat did not correlate with any of these markers, which is also supported in the literature [14,15,19]. Thus, this ectopic kidney fat depot seems also in the present study not be a metabolic relevant body fat depot.

The amount of kidney sinus fat has potential implications for the clinical practice. In the Framingham trial, individuals with high renal sinus fat showed a higher risk for hypertension and chronic kidney disease, even after adjusting for BMI and VAT [17]. Additionally, individuals with high renal sinus fat showed higher rates of microalbuminuria, which harbours an increased risk for major adverse cardiovascular events (MACE) and an elevated mortality for individuals with albuminuria [17,46,47,48]. This applied not only to a population with cardiovascular risk factors such as DM, but also to the general population [48]. Additionally, not only micro- and macroalbuminuria elevated the risk profile, but also a minimal increase in albuminuria, even within the normal values, increased the relative risk for MACE [49]. As the association of kidney sinus fat and hypertension as well as chronic kidney disease were independent of other fat depots, Foster et al. suggest a relevant role of kidney fat in blood pressure regulation and thus in the development of chronic kidney disease [17]. In this regard, kidney sinus fat is considered as a perivascular fat depot, which influences the vascular regulation and thus influences the blood flow, which again has an impact on the glomular function [50]. With an increase in pro-inflammatory signalling, the kidney sinus fat may have a negative impact on the kidney function and in the development of chronic kidney disease [50]. Thus, a reduction in albuminuria due to weight loss and KSF loss might not only improve chronic kidney disease, but may also reduce the risk for cardiovascular events [45,51].

Therefore, it is of great importance for disease prevention, that it is possible to decrease the amount of kidney sinus fat with weight loss. Furthermore, a moderate weight loss regime such as that in the present study with an average weight loss of “only” 5 kg is able to affect these changes which enable a benefit for the metabolic profile and disease prevention beyond the mere loss of 5 kg body weight [29].

In addition to weight loss, also drugs, which cause weight loss and lipolysis may show a reduction in KSF. For example, sodium–glucose co-transporter-2 (SGLT2)- inhibitors result in a negative energy balance and thus in weight loss via glucosuria [52]. The weight loss is firstly caused by depletion of glycogen of the liver and water loss. Later, the weight loss is caused by a depletion of adipose tissue depots including VAT, SAT and ectopic hepatic fat [52]. Thus, SGLT2 inhibitors may also cause a lipolysis of the kidney sinus fat, which could be analysed in future studies in patients eligible for SGLT2 inhibitors.

The strengths of this study are the availability of whole-body-MRI-based measurements of kidney fat, but also of VAT, SAT, LFC and PFC as well as a wide range of metabolic biomarkers over three time points including a follow-up time until week 50 after baseline to analyse long term effects after the dietary intervention, and the large number of included study participants.

The limitations to this study are as follows: only healthy overweight and obese individuals were included. Individuals with (chronic) kidney diseases or other chronic diseases such as diabetes or liver damage were excluded. Thus, the findings of this trial may not relate to patients with kidney or other chronic diseases. At the same time, the fact that our trial consisted of metabolically healthy overweight people, who did not have diabetes or use statins, may have been in advantage in that we were able to investigate the mere effects of calorie restriction without potential confounding effects by drugs such as SGLT-2 inhibitors, which may affect kidney fat content [36]. Another limitation of the present study is that the GFR was estimated, but not measured directly. Additionally, the perirenal fat within the Gerota’s fascia was not quantified. However, as this adipose tissue compartment is part of the retroperitoneal adipose tissue, it was measured as part of the visceral adipose tissue (VAT). A previous study by this group showed that the retroperitoneal adipose tissue seems to behave like the VAT [53].

## 5. Conclusions

In conclusion, the present study shows a significant decrease in kidney sinus fat, but not in kidney cortex fat content after 12 weeks of weight loss intervention, which was sustained after 50 weeks. After adjustment for change in VAT, the decrease in kidney sinus fat content remained significant. Accompanying this, a significant improvement in the GFR and creatinine was found after 12 weeks of intervention, which, however, did not sustain after 50 weeks. Kidney sinus fat correlated with body fat depots such as VAT, SAT, LFC and PFC, anthropometrics (BMI and waist circumference), markers of glucose, lipid and liver metabolism, and also with clinical markers such as diastolic blood pressure, while kidney cortex fat showed no significant correlation with these parameters. Thus, kidney sinus fat seems to be the metabolic relevant ectopic fat depot of the kidney, which explains known associations with obesity-related diseases and chronic kidney disease. Therefore, it is of great importance for disease prevention, that even a moderate weight loss enables a decrease in kidney sinus fat and thus a possible improvement in one’s metabolic profile.

## Figures and Tables

**Table 1:** Characteristics of the four weight loss groups (Q1 to Q4) at baseline, *n* = 137.

	Q1 (*n* = 35)	Q2 (*n* = 34)	Q3 (*n* = 35)	Q4 (*n* = 33)
	≤2%	>2% to ≤4.5%	>4.5% to ≤7.5%	>7.5%
Demographics				
Women (*n* (%))	19 (54.3)	14 (41.2)	18 (51.4)	17 (51.5)
Age (years)	50.9 ± 6.4	50.8 ± 8.4	51.2 ± 7.9	47.3 ± 8.5
Anthropometrics				
Waist circumference (cm)	106.1 ± 12.0	104.9 ± 10.7	103.1 ± 10.8	103.5 ± 12.2
BMI (kg/m^2^)	32.2 ± 4.1	30.8 ± 3.6	31.0 ± 3.4	31.6 ± 3.6
Blood pressure				
Systolic BP (mmHg)	139.5 ± 11.2	132.7 ± 14.2	136.5 ± 14.6	140.4 ± 22.8
Diastolic BP (mmHg)	90.0 ± 8.2	85.6 ± 8.2	87.2 ± 7.8	87.0 ± 10.2
Fat depots				
VAT (L)	5.3 ± 2.2	4.8 ± 2.1	4.8 ± 2.0	4.7 ± 2.1
SAT (L)	13.1 ± 4.6	11.2 ± 2.9	12.1 ± 3.9	12.9 ± 4.1
Total kidney fat content (%)	58.9 ± 18.5	52.3 ± 18.4	52.0 ± 16.8	52.8 ± 13.3
Kidney cortex fat content (%)	3.6 ± 1.8	3.2 ± 1.2	2.9 ± 1.2	3.4 ± 1.8
Kidney sinus fat content (%)	55.3 ± 18.3	49.2 ± 18.4	49.1 ± 16.8	49.4 ± 13.2
Liver fat content (%)	7.1 ± 4.4	8.8 ± 7.8	7.9 ± 6.5	7.4 ± 4.9
Liver function				
ALT (U/L)	25.0 ± 7.3	30.8 ± 13.9	26.6 ± 12.2	24.8 ± 9.9
AST (U/L)	21.7 ± 4.0	25.4 ± 6.8	22.3 ± 3.9	22.4 ± 5.2
GGT (U/L)	29.4 ± 14.0	25.5 ± 16.2	29.8 ± 19.7	24.3 ± 12.6
Lipid metabolism				
Triglycerides (mg/dL)	138.9 ± 65.7	135.0 ± 91.8	145.3 ± 94.2	109.4 ± 55.0
Cholesterol (mg/dL)	210.3 ± 33.9	201.9 ± 36.9	214.5 ± 36.5	203.3 ± 32.0
HDL (mg/dL)	53.5 ± 14.8	53.1 ± 14.5	56.3 ± 13.5	53.4 ± 15.3
LDL (mg/dL)	129.1 ± 26.2	120.2 ± 25.7	129.2 ± 26.8	128.0 ± 27.9
Glucose metabolism				
Glucose (mg/dL)	93.5 ± 8.0	93.2 ± 7.0	94.9 ± 6.9	91.9 ± 8.3
Insulin (mU/L)	14.9 ± 7.8	12.0 ± 6.9	10.9 ± 5.1	11.4 ± 5.6
HbA1c (%)	5.4 ± 0.4	5.5 ± 0.3	5.5 ± 0.3	5.5 ± 0.3
HOMA-IR	3.5 ± 1.9	2.8 ± 1.8	2.6 ± 1.2	2.6 ± 1.4
Kidney function				
Creatinine (mg/dL)	0.8 ± 0.1	0.8 ± 0.1	0.8 ± 0.1	0.8 ± 0.1
GFR (ml/min)	100.6 ± 7.0	99.4 ± 6.4	100.2 ± 12.6	103.1 ± 8.2
Albumin (g/L)	44.0 ± 2.0	43.7 ± 2.2	43.2 ± 2.2	43.7 ± 2.4
Inflammation				
CRP (ng/pL)	7.0 ± 8.7	4.1 ± 5.5	3.8 ± 2.8	3.9 ± 3.8
IFN-γ (ng/µL)	16.6 ± 16.1	12.9 ± 12.9	17.5 ± 16.7	11.1 ± 7.8
TNF-α (ng/µL)	4.3 ± 2.7	4.0 ± 2.5	5.0 ± 2.6	4.2 ± 2.5
IL-6 (ng/µL)	2.0 ± 1.7	1.8 ± 3.5	1.3 ± 0.8	1.3 ± 1.1
IL-8 (ng/µL)	10.6 ± 4.4	14.2 ± 23.7	9.8 ± 4.8	10.6 ± 5.3
LDH (U/L)	197.3 ± 30.1	197.4 ± 26.8	192.7 ± 31.8	200.2 ± 28.3
Adipokines				
Adiponectin (ng/mL)	15.6 ± 8.4	18.7 ± 11.4	16.9 ± 11.4	19.9 ± 13.7
Leptin (ng/mL)	29.2 ± 25.3	19.8 ± 20.3	21.5 ± 15.2	29.7 ± 29.4
Resistin (ng/mL)	5.7 ± 2.5	5.4 ± 2.1	5.3 ± 1.5	6.2 ± 3.3

Data are shown as mean ± SD. Abbreviations: ALT, alanine aminotransferase; AST, aspartat aminotransferase; BMI, body mass index; CRP, C-reactive protein; GFR, glomerular filtration rate; GGT, gamma glutamyl transferase; IFN-γ, interferon gamma; IL6, interleukin 6; IL8, interleukin 8; HbA1c, hemoglobin A1c; HDL, high density lipoprotein; HOMA-IR, homeostatic model assessment for insulin resistance; LDH, lactate dehydrogenase; LDL, low density lipoprotein, SAT, subcutaneous adipose tissue; TNF-α, tumour necrosis factor-alpha; VAT, visceral adipose tissue.

**Table 2:** Repeated measures correlations of kidney cortex fat, kidney sinus fat and kidney total fat over all time points (time points T0, T1 and T3 combined).

	Kidney Cortex			Kidney Sinus Fat			Total Kidney Fat		
	r_rm_	*p*	Adjusted *p*	r_rm_	*p*	Adjusted *p*	r_rm_	*p*	Adjusted *p*
Anthropometrics									
BMI	0.03	0.66	1.00	0.35	<0.0001 *	<0.0001 *	0.36	<0.0001 *	<0.0001 *
Waist	−0.02	0.73	1.00	0.26	<0.0001 *	<0.0001 *	0.26	<0.0001 *	<0.0001 *
Fat depots									
VAT	0.02	0.70	1.00	0.38	<0.0001 *	<0.0001 *	0.39	<0.0001 *	<0.0001 *
SAT	0.01	0.89	1.00	0.31	<0.0001 *	<0.0001 *	0.33	<0.0001 *	<0.0001 *
Liver fat content	0.00	0.96	1.00	0.32	<0.0001 *	<0.0001 *	0.32	<0.0001 *	<0.0001 *
Pancreas fat content	0.02	0.76	1.00	0.07	0.25	0.74	0.08	0.19	0.58
Blood pressure									
Systolic BP	0.05	0.41	1.00	0.07	0.26	0.78	0.08	0.20	0.60
Diastolic BP	0.01	0.93	1.00	0.15	0.01 *	0.04 *	0.16	0.01 *	0.03 *
Adipokine									
Leptin	0.02	0.75	1.00	0.23	<0.001 *	<0.001 *	0.24	<0.0001 *	<0.001 *
Adiponectin	−0.01	0.88	1.00	0.02	0.79	1.00	0.03	0.76	1.00
Resistin	−0.16	0.01 *	0.03 *	0.01	0.84	1.00	−0.02	0.80	1.00
Glucose metabolism									
HOMA-IR	0.05	0.38	1.00	0.12	0.06	0.17	0.13	0.04 *	0.11
Insulin	0.06	0.37	1.00	0.10	0.12	0.36	0.11	0.08	0.25
Glucose	0.01	0.87	1.00	0.19	0.002 *	0.01 *	0.20	<0.002 *	<0.004 *
IGF-1	0.02	0.70	1.00	−0.11	0.07	0.21	−0.12	0.06	0.18
HbA1c	−0.07	0.26	0.77	0.11	0.08	0.24	0.11	0.09	0.27
Lipid metabolism									
Triglycerides	0.00	0.97	1.00	0.13	0.04 *	0.12	0.14	0.03 *	0.08
Cholesterol	−0.05	0.42	1.00	0.23	<0.001 *	<0.001 *	0.23	<0.001 *	<0.001 *
LDL	−0.08	0.21	0.64	0.16	0.01 *	0.03 *	0.15	0.02 *	0.06
HDL	−0.01	0.85	1.00	0.18	<0.003 *	0.01 *	0.18	<0.003 *	0.01 *
Liver function tests									
ALT	0.04	0.55	1.00	0.05	0.42	1.00	0.07	0.24	0.72
AST	0.00	0.97	1.00	0.01	0.89	1.00	0.02	0.73	1.00
GGT	−0.01	0.92	1.00	0.31	<0.0001 *	<0.0001 *	0.32	<0.0001 *	<0.0001 *
Inflammation									
CRP	0.01	0.90	1.00	0.09	0.16	0.49	0.09	0.13	0.39
TNF α	−0.02	0.77	1.00	−0.03	0.68	1.00	−0.03	0.66	1.00
IFN	−0.04	0.50	1.00	−0.02	0.76	1.00	−0.02	0.69	1.00
IL6	−0.05	0.45	1.00	−0.01	0.88	1.00	−0.02	0.79	1.00
IL8	−0.13	0.04 *	0.11	0.00	1.00	1.00	−0.01	0.88	1.00
LDH	−0.01	0.92	1.00	0.21	0.02 *	0.05 *	0.23	0.01 *	0.03 *
Kidney function tests									
Creatinine	−0.07	0.25	0.76	−0.05	0.38	1.00	−0.08	0.22	0.66
GFR	0.06	0.33	0.99	0.03	0.58	1.00	0.05	0.42	1.00
Albumin	0.01	0.90	1.00	0.19	<0.002 *	0.01 *	0.20	<0.001 *	<0.003 *

* *p* values were adjusted using Bonferroni correction, on the *p*-values generated for each bioclinical variables with all three kidney fat parameters. Thus, only *p*-values less than 0.0167, i.e., (0.05/3) remained significant after adjustment. Abbreviations: ALT, alanine aminotransferase; AST, aspartate aminotransferase; BP, blood pressure; BMI, body mass index; CRP, C-reactive protein; GFR, glomerular filtration rate; GGT, gamma glutamyl transferase; IFN-γ, interferon gamma; IL6, interleukin 6; IL8, interleukin 8; HbA1c, hemoglobin A1c; HDL, high density lipoprotein; HOMA-IR, homeostatic model assessment for insulin resistance; LDH, lactate dehydrogenase; LDL, low density lipoprotein, SAT, subcutaneous adipose tissue; TNF-α, tumour necrosis factor-alpha; VAT, visceral adipose tissue.

**Table 3:** Relative changes of body weight, total kidney fat content, renal cortex fat and sinus fat content, creatinine and glomerular filtration rate (GFR) from baseline to week 12 and from baseline to week 50; *n* = 137.

		Baseline	Week 12	Log_e_ Relative Change	*p*	Week 50	Log_e_ Relative Change	*p*
		Mean ± SD	Mean ± SD	(Week 12)		Mean ± SD	(Week 50)	
Weight (kg)	Q1	94.8 ± 15.7	94.8 ± 15.6	0.0 ± 0.2	<0.0001 *	96.1 ± 16.1	1.3 ± 0.6	<0.0001 *
	Q2	93.7 ± 14.4	90.7 ± 14.0	−3.3 ± 0.1		93.3 ± 13.9	−1.3 ± 0.5	
	Q3	93.9 ± 15.3	88.4 ± 14.4	−6.1 ± 0.2		89.8 ± 15.8	−4.4 ± 0.8	
	Q4	94.7 ± 14.4	84.4 ± 12.8	−11.5 ± 0.6		84.8 ± 14.2	−11.2 ± 1.6	
Total kidney fat content (%)	Q1	58.9 ± 18.5	54.9 ± 18.6	−6.8 ± 4.4	0.009 *	57.4 ± 19.5	−3.7 ± 3.8	0.002 *
	Q2	52.3 ± 18.4	49.8 ± 17.1	−5.5 ± 4.0		55.2 ± 17.6	6.7 ± 3.5	
	Q3	52.0 ± 16.8	46.8 ± 16.0	−13.2 ± 5.5		49.2 ± 18.4	−8.4 ± 5.1	
	Q4	52.8 ± 13.3	43.7 ± 13.4	−21.3 ± 5.8		45.5 ± 16.5	−21.4 ± 5.0	
Kidney cortex fat content (%)	Q1	3.6 ± 1.8	3.4 ± 1.5	−5.0 ± 10.0	0.97	3.5 ± 1.7	−7.5 ± 11.9	0.77
	Q2	3.2 ± 1.2	3.1 ± 1.1	0.2 ± 8.3		3.3 ± 1.7	3.1 ± 10.6	
	Q3	2.9 ± 1.2	3.3 ± 1.8	11.7 ± 11.2		2.6 ± 1.1	−10.5 ± 11.3	
	Q4	3.4 ± 1.8	2.9 ± 1.8	−15.7 ± 11.6		2.9 ± 1.0	−9.4 ± 9.2	
Kidney sinus fat content (%)	Q1	55.3 ± 18.3	51.5 ± 18.3	−6.4 ± 5.1	0.02 *	53.9 ± 19.5	−3.2 ± 4.8	0.001 *
	Q2	49.2 ± 18.4	46.7 ± 16.9	−5.4 ± 4.2		52.0 ± 17.3	7.3 ± 3.8	
	Q3	49.1 ± 16.8	43.4 ± 16.1	−15.4 ± 6.3		46.5 ± 18.5	−8.6 ± 5.7	
	Q4	49.4 ± 13.2	40.7 ± 13.0	−21.3 ± 6.3		42.7 ± 16.1	−22.0 ± 5.4	
Creatinine	Q1	0.783 ± 0.133	0.809 ± 0.136	2.8 ± 1.4	0.02 *	0.805 ± 0.131	3.0 ± 1.6	0.77
	Q2	0.831 ± 0.125	0.84 ± 0.127	2.0 ± 1.3		0.833 ± 0.134	−1.1 ± 1.2	
	Q3	0.771 ± 0.122	0.795 ± 0.108	3.4 ± 1.3		0.809 ± 0.144	4.8 ± 1.4	
	Q4	0.789 ± 0.103	0.773 ± 0.132	−2.5 ± 1.8		0.815 ± 0.107	2.2 ± 2.0	
GFR	Q1	100.6 ± 6.9	99.6 ± 7.2	−1.0 ± 0.5	0.04 *	99.6 ± 6.7	−1.0 ± 0.6	0.65
	Q2	99.2 ± 6.2	98.5 ± 5.9	−0.8 ± 0.5		99.8 ±7.1	0.8 ± 0.7	
	Q3	100.3 ± 12.4	99.1 ± 10.1	−0.8 ± 0.8		97.9 ± 12.3	−2.1 ± 0.7	
	Q4	103.1 ± 8.0	104.3 ± 8.7	1.1 ± 0.7		102.3 ± 7.3	−0.8 ± 0.8	

* *p* = significant at the 0.05 level (two tailed). Data are shown as mean ± SD and as mean ± SEM for week 12 and week 50 for Loge relative change with baseline values as reference. *p*-values of the four weight loss quartiles were calculated with linear mixed models adjusted for sex and age (baseline to week 12, baseline to week 50). Abbreviations: GFR, glomerular filtration rate; SD, standard deviation; SEM, standard error of the mean.

## Data Availability

All data is available from the corresponding author on request based on a data transfer agreement, which is required according to the informed consent statement used for the study.

## Supplementary Materials

The following supporting information can be downloaded at: https://www.mdpi.com/article/10.3390/nu14071437/s1, Table S1: Spearman’s correlations between anthropometric parameters, fat depositions, metabolic parameters, blood pressure, and kidney fat content at baseline; *n* = 137.
