# Supplementary material for: Changes in Kidney Fat upon Dietary-Induced Weight Loss
Source: Nutrients. 2022 Mar 30;14(7):1437. doi: 10.3390/nu14071437 (PMC9002806; doi:10.3390/nu14071437)
Supplement: Supplementary file 1 [file nutrients-14-01437-s001.zip › nutrients-1647885-supplementary.pdf]

Correlations of kidney fat content in comparison to blood biomarkers, blood pressure, anthropometrics and body fat depositions

Spearman`s correlations of total kidney fat content, renal cortex fat content and renal sinus fat content with weight, waist circumference, BMI, systolic and diastolic blood pressure, VAT, SAT, hepatic and pancreatic fat content, and blood biomarkers at baseline, after 12 and 50 weeks (for each time point separately) are shown in table S1.

By using the Spearman`s correlations, we found strong correlations with rho > 0.4 and p < 0.05 between total kidney fat and waist circumference after 50 weeks with rho = 0.41 and p <.0001. Similar to total kidney fat, sinus fat showed a strong correlation with waist circumference after 50 weeks with rho = 0.4 and p <.0001. Between total kidney fat and VAT we also observed strong correlations (rho > 0.4 and p < 0.05) at baseline, after 12 weeks and after 50 weeks with rho = 0.46 and p <.0001 at baseline, rho = 0.46 and p <.0001 after 12 weeks, and rho = 0.56 and p <.0001 after 50 weeks, which we also observed between renal sinus fat and VAT at all three time points with rho = 0.45 and p <.0001 at baseline, rho = 0.44 and P <.0001 after 12 weeks, and with rho = 0.55 and p <.0001 after 50 weeks. For renal cortex fat, we could not observe these strong correlations.

Weaker correlations between total kidney fat and waist circumference were observed at baseline and after 12 weeks with rho < 0.4 and p < 0.05. The same was seen for sinus fat and waist circumference at these time points and between cortex fat and waist circumference at all three time points. Weaker correlations with rho < 0.4 and p <0.05 at all three time points (baseline, week 12, week 50) were observed between total kidney fat and liver fat, pancreatic fat, GGT, triglycerides, insulin, HOMA-IR, CRP, and leptin as well as between sinus fat and all these blood parameters, i.e. the correlations for total kidney fat and sinus fat were in parallel to each other. For renal cortex fat, a weak correlation with rho < 0.4 and p <.005 at all three time points was observed only for VAT. BMI correlated with total kidney fat, cortex fat, and sinus fat after 12 weeks and after 50 weeks, but not at baseline. SAT correlated with cortex fat and with sinus fat at week 12 and at week 50 with rho < 0.4 and p <0.05. Weight showed a weaker correlation with total kidney fat and sinus fat after 12 weeks and after 50 weeks with rho < 0.4 and p < 0.05. Diastolic blood pressure correlated at baseline and after 50 weeks with total kidney fat and sinus fat with rho < 0.4 and p < 0.05. Other parameters (ALT, HDL, IL-6 with total kidney fat content; weight, diastolic blood pressure, liver fat content, LDL, glucose, CRP with cortex fat; and ALT, HDL, IL-6 with renal sinus fat) showed individual weak correlations with rho < 0.04 and p < 0.05 without any pattern.

Table S1. Spearman’s correlations between anthropometric parameters, fat depositions, metabolic parameters, blood pressure, and kidney fat content at baseline; *n* = 137.

Total kidney fat content						
	Baseline		Week 12		Week 50	
	rho	p-value	rho	p-value	rho	p-value
Weight	0.14	0.09	0.24	0.00*	0.36	<.0001*
Waist circumference	0.21	0.01*	0.32	0.00*	0.41	<.0001*
BMI	0.12	0.14	0.23	0.01*	0.34	0.00*
Systolic blood pressure	0.09	0.30	0.07	0.42	0.17	0.06
Diastolic blood pressure	0.16	0.05*	0.13	0.13	0.26	0.00*
**Fat depositions**						
VAT	0.46	<.0001*	0.46	<.0001*	0.56	<.0001*
SAT	0.14	0.09	0.32	0.00*	0.31	0.00*
Liver Fat Content	0.24	0.00*	0.29	0.00*	0.37	<.0001*
Pancreatic fat content	0.27	0.00*	0.31	0.00*	0.33	0.00*
**Liver function tests**						
ALT	0.13	0.12	0.21	0.02*	0.10	0.28
AST	-0.13	0.13	-0.05	0.56	-0.02	0.83
GGT	0.16	0.05*	0.24	0.01*	0.19	0.03*

**Lipid metabolism**						
Triglycerides	0.2	0.02*	0.26	0.00*	0.24	0.01*
Cholesterol	0.03	0.70	0.04	0.62	0.02	0.86
HDL	-0.16	0.06	-0.16	0.07	-0.26	0.00*
LDL	0.00	0.99	0.04	0.66	0.01	0.92
**Glucose metabolism**						
Glucose	0.13	0.13	0.12	0.17	0.24	0.01*
Insulin	0.21	0.01*	0.19	0.03*	0.25	0.01*
HbA1c	0.13	0.12	0.13	0.14	0.16	0.08
HOMA-IR	0.21	0.01*	0.20	0.03*	0.28	0.00*
**Kidney function tests**						
Creatinine	-0.05	0.56	0.00	0.97	0.05	0.61
GFR	0.14	0.11	0.04	0.67	-0.01	0.90
Albumin	-0.08	0.33	-0.14	0.12	-0.05	0.62
**Inflammation**						
CRP	0.16	0.05*	0.27	0.00*	0.24	0.01*
INF- γ	-0.12	0.17	-0.10	0.25	0.11	0.23
TNF-α	-0.14	0.10				
IL-6	0.00	1.00	0.00	0.98	0.22	0.02*
IL-8	0.02	0.83	-0.05	0.57	-0.04	0.65
LDH	0.05	0.53	0.10	0.27	-	-
**Adipokine**						
Adiponectin	0.06	0.49				
Leptin	0.18	0.04*	0.29	0.00*	0.27	0.00*
Resistin	0.02	0.83				

	**Renal cortex fat content**					
	Baseline		Week 12		Week 50	
	rho	p-value	rho	p-value	rho	p-value
Weight	0.25	0.00*	0.17	0.06	0.15	0.10
Waist circumference	0.17	0.04*	0.19	0.03*	0.17	0.06
BMI	0.14	0.11	0.21	0.02*	0.18	0.04*
Systolic blood pressure	0.03	0.70	0.02	0.81	0.20	0.02*
Diastolic blood pressure	0.05	0.56	0.07	0.44	0.24	0.01*
**Fat depositions**						
VAT	0.28	0.00*	0.26	0.00*	0.28	0.00*
SAT	0.12	0.17	0.27	0.00*	0.19	0.04*
Liver Fat Content	0.09	0.26	0.15	0.09	0.22	0.02*
Pancreatic fat content	0.00	0.97	0.18	0.04*	0.06	0.52
**Liver function tests**						
ALT	-0.02	0.81	0.11	0.22	0.03	0.73
AST	-0.06	0.45	-0.11	0.20	-0.11	0.22
GGT	-0.03	0.73	0.08	0.36	0.07	0.43
**Lipid metabolism**						
Triglycerides	0.07	0.43	0.08	0.39	0.15	0.11
Cholesterol	0.11	0.18	0.09	0.28	0.02	0.86
HDL	-0.12	0.15	-0.06	0.53	-0.04	0.65
LDL	0.21	0.01*	0.12	0.18	-0.03	0.74

**Glucose metabolism**						
Glucose	0.07	0.39	0.19	0.03*	-0.08	0.38
Insulin	0.23	0.01*	0.13	0.16	0.20	0.02*
HbA1c	-0.02	0.83	0.01	0.89	0.01	0.89
HOMA-IR	0.22	0.01*	0.15	0.10	0.18	0.05*
**Kidney function tests**						
Creatinine	0.07	0.39	-0.04	0.62	-0.02	0.79
GFR	-0.08	0.37	0.06	0.48	0.05	0.56
Albumin	-0.11	0.21	-0.17	0.06	-0.07	0.43
**Inflammation**						
CRP	-0.01	0.93	0.10	0.27	0.20	0.02*
INF- γ	0.00	0.96	-0.16	0.06	0.06	0.52
TNF-α	0.05	0.54				
IL-6	0.10	0.26	0.05	0.58	0.09	0.35
IL-8	0.08	0.32	-0.06	0.49	-0.04	0.69
LDH	0.10	0.26	-0.07	0.42	-	-
**Adipokine**						
Adiponectin	-0.06	0.46				
Leptin	0.16	0.07	0.27	0.00*	0.22	0.01*
Resistin	-0.13	0.11				

**Renal sinus fat content**						
	Baseline		Week 12		Week 50	
	rho	p-value	rho	p-value	rho	p-value
Weight	0.12	0.16	0.23	0.01*	0.36	<.0001*
Waist circumference	0.20	0.02*	0.30	0.00*	0.40	<.0001*
BMI	0.11	0.19	0.21	0.02*	0.33	0.00*
Systolic blood pressure	0.08	0.34	0.07	0.44	0.15	0.09
Diastolic blood pressure	0.17	0.05*	0.13	0.14	0.24	0.01*
**Fat depositions**						
VAT	0.45	<.0001*	0.44	<.0001*	0.55	<.0001*
SAT	0.13	0.12	0.28	0.00*	0.29	0.00*
Liver Fat Content	0.23	0.01*	0.29	0.00*	0.35	<.0001*
Pancreatic fat content	0.27	0.00*	0.29	0.00*	0.33	0.00*
**Liver function tests**						
ALT	0.14	0.11	0.18	0.04*	0.09	0.32
AST	-0.13	0.14	-0.05	0.54	-0.01	0.88
GGT	0.18	0.04*	0.22	0.01*	0.19	0.03*
**Lipid metabolism**						
Triglycerides	0.20	0.02*	0.27	0.00*	0.24	0.01*
Cholesterol	0.03	0.76	0.04	0.65	0.01	0.88
HDL	-0.16	0.07	-0.14	0.11	-0.27	0.00*
LDL	-0.01	0.90	0.03	0.75	0.01	0.88
**Glucose metabolism**						
Glucose	0.12	0.14	0.11	0.20	0.26	0.00*
Insulin	0.20	0.02*	0.18	0.04*	0.24	0.01*
HbA1c	0.13	0.14	0.13	0.13	0.17	0.06
HOMA-IR	0.20	0.02*	0.18	0.04*	0.27	0.00*
**Kidney function tests**						

Creatinine	-0.05	0.52	0.01	0.93	0.06	0.54
GFR	0.14	0.09	0.03	0.72	-0.02	0.79
Albumin	-0.07	0.43	-0.12	0.18	-0.05	0.57
**Inflammation**						
CRP	0.17	0.05*	0.26	0.00*	0.24	0.01*
INF- γ	-0.12	0.15	-0.08	0.35	0.10	0.26
TNF-α	-0.14	0.10				
IL-6	0.00	0.96	-0.01	0.89	0.21	0.02*
IL-8	0.01	0.92	-0.06	0.51	-0.04	0.65
LDH	0.05	0.59	0.09	0.29	-	-
**Adipokine**						
Adiponectin	0.06	0.51				
Leptin	0.17	0.05*	0.27	0.00*	0.26	0.00*
Resistin	0.02	0.77				

* Correlation is significant at the 0.05 level (two tailed). Abbreviations: ALT, alanine aminotransferase; AST, aspartat aminotransferase; BMI, body mass index; CRP, C-reactive protein; GFR, glomerular filtration rate; GGT, gamma glutamyl transferase; IFN-γ, interferon gamma; IL6, interleukin 6; IL8, interleukin 8; HbA1c, hemoglobin A1c; HDL, high density lipoprotein; HOMA-IR, homeostatic model assessment for insulin resistance; LDH, lactatdehydrogenase; LDL, low density lipoprotein, SAT, subcutaneous adipose tissue; TNF-α, tumor necrosis factor-alpha; VAT, visceral adipose tissue
